# ERRα Regulates OTUB1 Expression to Promote Colorectal Cancer Cell Migration

**DOI:** 10.7150/jca.30720

**Published:** 2019-10-06

**Authors:** Yi Zhou, Qingzhu Jia, Xiaoqing Meng, Diangang Chen, Bo Zhu

**Affiliations:** 1 Institute of Cancer, Xinqiao Hospital, Third Military Medical University, Chongqing, China; 2 Department of Hematology, the Third Affiliated Hospital of Chongqing Medical University (Gener Hospital), Chongqing, China

**Keywords:** OTUB1, ERRα, colorectal cancer, transcriptional regulation, cell migration

## Abstract

Ovarian tumor domain-containing ubiquitin aldehyde binding protein 1 (OTUB1) is overexpressed in many cancers and plays an important role in tumor progression and metastasis. However, the molecular mechanisms underlying OTUB1 overexpression are not clear. In this study, we found that estrogen-related receptor alpha (ERRα, also called NR3B1) binds to *OTUB1* promoter and regulates its expression in colorectal cancer. Furthermore, ERRα promoted the migration of CRC cells by inducing vimentin expression via OTUB1. Our data show that OTUB1 is a novel target of ERRα and indicate that ERRα-OTUB1 signaling may play a significant role in CRC metastasis.

## Introduction

Colorectal cancer (CRC) is the fifth most common cancer in China: in 2015, the incidence of CRC in China was about 376,300 new cases, and the mortality was 191,000 cases 1. The primary factor causing death in patients with CRC is metastasis. Therefore, understanding the metastatic mechanisms in CRC is important for improving treatment strategies.

Estrogen-related receptor alpha (ERRα, also called NR3B1), encoded by the *ESRRA* gene, is a member of the ligand-independent orphan nuclear receptor superfamily 2. ERRα shares high sequence identity with ERα, but a genome-wide binding site location analysis indicated that most of the genes regulated by ERRα are distinct from those regulated by ERα 3. ERRα regulates the expression of genes related to cell metabolism, such as those involved in the tricarboxylic acid cycle, aerobic glycolysis, and lipogenesis 4-7. However, recent studies have shown that ERRα might not only regulate metabolism, but might also participate in the proliferation and metastasis of cancer cells. For example, ERRα promotes cellular migration by activating the expression of TNFAIP1 and subsequently destabilizing RHOA 8. The activity of ERRα is associated with the WNT signal. In this regard, a study has shown that transcriptional upregulation of WNT11 by ERRα influences the migration of cancer cells 9. ERRα plays an important role in the development of hormone-dependent cancers. ERRα levels are higher in breast, ovarian, cervical and prostate cancer tissues than in normal tissues, and patients with high ERRα expression have poor survival 10-13. Moreover, ERRα promotes cancer proliferation and metastasis in non-hormone-dependent tumors, such as oral squamous cell carcinoma 14. In CRC, the mRNA levels of *ERRα* are higher in tumor tissues than in normal mucosa, and increase significantly from TNM stage II to IV 15. Furthermore, high ERRα expression is significantly associated with an increased risk of recurrence and poor prognosis in CRC 16.

Ovarian tumor domain-containing ubiquitin aldehyde binding protein 1 (OTUB1) is a deubiquitinating enzyme that belongs to the OTU family of cysteine proteases. By specifically regulating Lys48-linked ubiquitin and Lys63-linked polyubiquitin, OTUB1 can target and inhibit E2 enzymes, such as UBC13, UBE2D, and UBE2E. OTUB1 regulates naive CD4+ T cell proliferation via GRAIL 17. OTUB1 is also a regulator of p53: by suppressing MDM2-mediated p53 ubiquitination, it stabilizes and activates p53, induces cell apoptosis and inhibits cell proliferation 18. Additionally, by stabilizing MDMX expression, OTUB1 enhances p53 phosphorylation at S46 and promotes the mitochondria-mediated apoptotic pathway 19. Recent studies have shown that OTUB1 plays a crucial role in tumorigenesis and cancer progression. By deubiquitinating and stabilizing phospho-SMAD2/3, induced by TGFβ, OTUB1 efficiently regulates TGFβ-mediated cell migration 20. By cleaving K48-linked polyubiquitin chains, OTUB1 regulates c-IAP1 expression and TWEAK-induced activation of canonical NF-κB and MAPK signaling pathways and modulates TNF-dependent cell death 21. We have previously shown that OTUB1 is overexpressed in CRC and high OTUB1 expression correlates with short survival 22. In this study, we evaluated the association between ERRα and OTUB1 expression in CRC and elucidated the mechanism through which ERRα regulated OTUB1 expression to promote CRC migration.

## Materials and Methods

### Cell lines and culture conditions

The human colon cancer cell lines HCT116 and Caco2 were obtained from the American Type Culture Collection. HCT116 cells were cultured in RPMI 1640 supplemented with 10% fetal bovine serum (FBS), and Caco2 cells were cultured in Dulbecco's modified Eagle's medium (DMEM) supplemented with 10% FBS. All cells were maintained in a humidified 5% CO_2_ atmosphere at 37 °C.

### Cell transfection

The pcDNA3.1-OTUB1 plasmid was generated amplifying OTUB1 from HCT116 using the primers 5′-ATTGGATCCACCATGGCGGCGGAGGAACCT- 3′ and 5′-ATGCTCGAGCTATTTGTAGAGGATATCGTA-3′. The amplified DNA was digested with BamHI and XhoI and inserted into the pcDNA3.1 plasmid.

The pcDNA3.1-ERRα plasmid was generated for amplifying ERRα from HCT116 using the primers 5′-TAGGATCCACCATGTCCAGCCAGGTGGTG-3′ and 5′-GCGAATTCTCAGTCCATCATGGCCTC-3′. The amplified DNA was digested with BamHI and EcoRI and the released fragment was inserted into the pcDNA3.1 plasmid.

The OTUB1 siRNA sequences were #1 (5′-UUAACTGUCUGGCCUAUGATT-3′) and #2 (5′-CCAUGUGCAAGGAGAGCGATT-3′) and the negative control (NC) siRNA was 5′-UUCUCCGAACGUGUCACGUTT-3′. The ERRα siRNA sequences were #1 (5′-UGGUGGGCAUUGAGCCUCUCUACAU-3′) and #2 (5′-GAAUGCACUGGUGUCUCAUCUGCUG-3′) and 5′-GGUAGGUGAGUGUACAGACGCAAUA-3' was the NC siRNA 9.

For transient transfections, 6 × 10^5^ HCT116 or 4 × 10^5^ Caco2 cells were seeded in 6-well plates. Twenty-four hours later, the cells were transfected with 4 μg of plasmid DNA or 100 nM siRNA using Lipofectamine^TM^ 2000 (Invitrogen) according to the manufacturer's protocol. After 48 h, the cells were collected for quantitative PCR (qPCR), western blotting, fluorescence, Chromatin Immunoprecipitation (CHIP) and migration assays.

### Western blot analysis

Total cellular proteins were extracted in lysis buffer (1% Triton X-100, 0.1% sodium dodecyl sulfate [pH 7.3], 50 mM Tris, and 150 mM NaCl) with protease inhibitors (Roche) for 30 min on ice and centrifuged at 16,000 *g* at 4 °C. Western blots were carried out as previously described 23. The primary antibodies used included anti-OTUB1, anti-ERRα, anti-β-actin, anti-vimentin, anti-zo-1, anti-snail, anti-E-cadherin and anti-β-catenin antibodies. Anti-OTUB1 antibody was purchased from abcom and the other antibodies were purchased from Cell Signaling Technology and used 1:1,000.

### Migration assays

To investigate the effect of ERRα and OTUB1 on the migration of colorectal cells, 6 × 10^5^ HCT116 or 4 × 10^5^ Caco2 cells were seeded in 6-well plates. Twenty-four hours later, the cells were transfected with 4 μg of pCDNA3.1-ERRα plasmid DNA and 100 nM OTUB1 siRNA or 100 nM ERRα siRNA and 4 μg pCDNA3.1-OTUB1 plasmid. After 24 h, 0.5 × 10^5^ HCT116 cells or 1.5 × 10^5^ Caco2 in serum-free medium were seeded into a Boyden chamber (8-μm pore; BD Falcon) to assay cell migration. The chambers were inserted in 24-well plates with medium with 10% FBS and incubated for 24 to 36 h at 37 °C with 5% CO_2_. The cells on the underside of filter membrane were fixed in ethanol, stained with crystal violet and counted under a microscope (Leica Biosystems ADMI300B).

### qPCR analysis of *OTUB1* expression

Total RNA was extracted from CRC cell lines using RNAiso Plus (Takara). Two micrograms of total RNA were used to synthesize complimentary DNA (cDNA) with the PrimeScript™ RT reagent Kit with gDNA Eraser (Takara), following the manufacturer's protocol. For qPCR, cDNA products were amplified using a SYBR Green PCR Kit (Takara). Quantification was performed using the 7500 real time PCR system (Applied Biosystems). *OTUB1* expression values were normalized to *β-actin* and calculated using the comparative CT method (2^-ΔΔCT^). The primer sequences for *OTUB1* were 5′-TTTCTATCGGGCTTTCGGA-3′ (forward) and 5′-TCGGAGGTGCTCTGGTCAT-3′ (reverse). The primer sequences for *β-actin* were 5′-TGGCACCCAGCACAATGAA-3′ (forward) and 5′-CTAAGTCATAGTCCGCCTAGAAGCA-3′ (reverse).

### Luciferase assays

The 2 kb -1,000/+1,000 *OTUB1* promoter sequence was amplified by PCR using HCT116 genomic DNA with the primer pair 5′-TATACGCGTAAACCAGAAGGACACAGA-3′ and 5′- TCCAGATCTAGGCGTGGTCTCTTGCAG-3′, digested with MluI and BgI II, and cloned into the MluI/Bgl II site of the pGL3-basic vector (Promega) to generate the pGL3-OTUB1 plasmid. The constructs containing the mutated *OTUB1* promoter sequence were generated by PCR using the -1,000/+1,000 OTUB1 promoter sequence DNA as template and the primers listed in Table S1.

HCT116 or Caco2 cells (4 × 10^4^) were seeded in 24-well plates. Twenty-four hours later, the cells transfected with 200 ng pGL3-OTUB1 promoter or pGL3 Basic, 10 ng pGL4.73, 200 ng pCDNA3.1-ERRα or pCDNA3.1, 10 nM ERRα siRNA or siNC, or 10 μM XCT790 or DMSO. After 48 h, the cells were washed by PBS and solubilized with Passive Lysis Buffer (Promega), then assayed for both Firefly and Renilla luciferase activities using a Dual-Luciferase Reporter Assay System (Promega) according to the manufacturer's instructions.

### ChIP

ChIP assays were performed using the SimpleCHIP^®^ enzymatic chromatin immunoprecipitation kit (Cell Signaling Technology, #9002) according to the manufacturer's protocol. Briefly, 12 × 10^6^ cells were cross-linked with 1% formaldehyde for 10 min at room temperature. Nuclei were isolated upon cells lysis and chromatin was digested into fragments of 150-900 bp by micrococcal nuclease. The nuclear membrane was disrupted by sonication. For immunoprecipitation, 5-10 μg of total chromatin were incubated overnight at 4 °C with 10 μg anti-ERRα antibodies (Cell Signaling Technology, #13826) or normal Rabbit IgG as negative control (Cell Signaling Technology, #2729). The antibody-DNA complexes were incubated with 30 μL of ChIP-grade protein G-agarose beads for 2 h at 4 °C, eluted, digested with Proteinase K for 2 h at 65 °C, and finally purified with spin columns. ERRα binding site on *OTUB1* promoter was assessed by qPCR using the primers 5′-CTCTAAGAAAGGCGAGGGT-3′ (forward) and 5′-CTCTTGCGGTCGTGGATA-3′ (reverse).

### Statistical analysis

Data were analyzed using the GraphPad Prism software V5.0 (Graphpad, San Diego, CA, USA). The Pearson's test was used for the correlation analysis. Statistical significance was determined using the two-tailed t-test, and P-values < 0.05 were considered significant.

## Results

### ERRα regulates OTUB1 expression

To explore the mechanisms underlying OTUB1 overexpression in CRC, we searched for transcription factors that may bind to the *OTUB1* promoter (and thereby regulate *OTUB1* expression) in the JASPAR database (http://jaspar.genereg.net). The correlation between *OTUB1* expression and these transcription factors was analyzed using the TCGA database. We found that the expression of ERRα significantly correlated with that of *OTUB1* in CRC (Figure S1).

**Figure 1:**
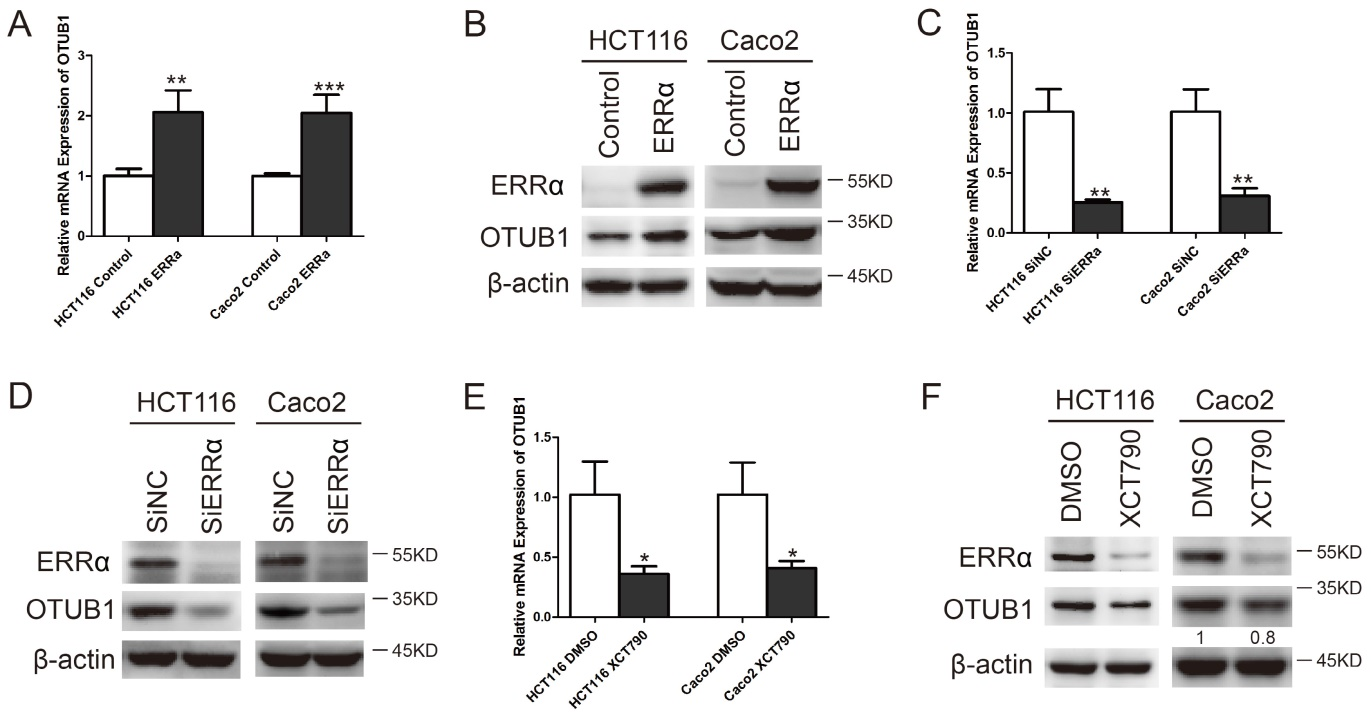
** ERRα regulates OTUB1.** HCT116 or Caco2 were transfected with an empty vector or a plasmid overexpressing ERRα, treated with DMSO or XCT790 (10 μM), or transfected with siNC or ERRα siRNA for 48 h. *OTUB1* mRNA levels in HCT116 and Caco2 were measured by q-PCR (A, C, E); ERRα and OTUB1 protein levels in HCT116 and Caco2 cells were measured by western blot (B, D, F). Data were normalized to β-actin. The data represent the mean of three independent experiments, and the error bars represent the SD. *, P < 0.05; **, P < 0.01; ***, P < 0.001.

To investigate whether OTUB1 expression is regulated by ERRα in CRC cells, we overexpressed or silenced ERRα or used the ERRα inhibitor XCT790. After transfecting HCT116 or Caco2 cells with an ERRα expression plasmid for 48 h, ERRα was found to be overexpressed (Figure 1B). Meanwhile, the levels of OTUB1 mRNA (Figure 1A) and protein (Figure 1B) were also increased. Two siRNAs were used to knockdown ERRα expression (Figure S2). We silenced or inhibited ERRα in HCT116 or Caco2, and western blot analysis showed that, in these cells, ERRα was downregulated (Figure 1D and F) and the levels of OTUB1 mRNA (Figure 1C and E) and protein (Figure 1D and F) were decreased. These results showed that overexpression or knockdown of ERRα significantly enhanced or suppressed OTUB1 expression.

### ERRα regulates *OTUB1* promoter activity

**Figure 2:**
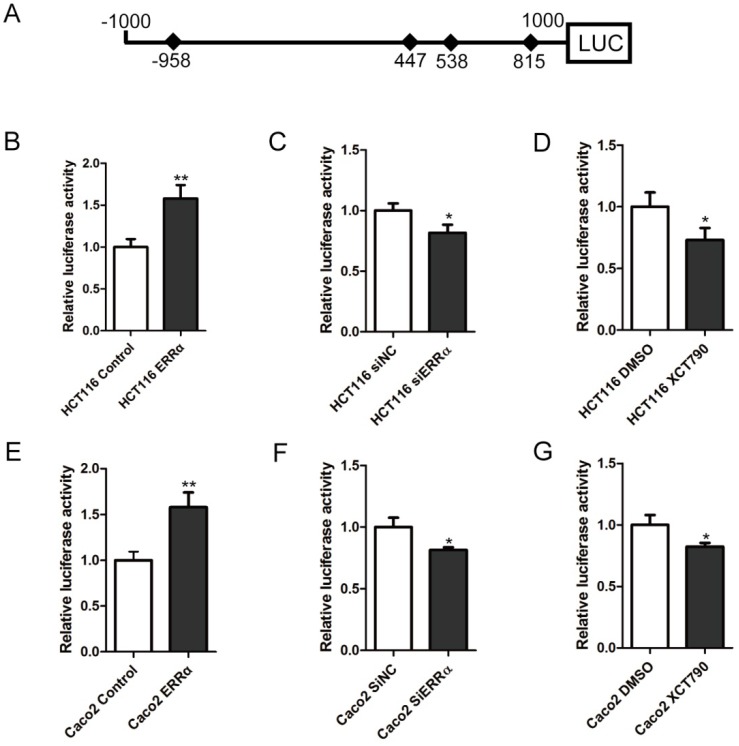
** ERRα induces *OTUB1* promoter activity.** (A) Scheme representing the cloning of *OTUB1* promoter sequence (-1000 to +1000) into the pGL3 reporter. pGL3 or pGL3-*OTUB1* were co-transfected with empty vector or an ERRα expression plasmid, DMSO or XCT790 (10 μM), siNC or ERRα siRNA for 48 h. The luciferase activity in HCT116 (B, C, D) or Caco2 (E, F, G) cells was then measured. Data were normalized to Renilla activity. The data represent the mean of three independent experiments, and the error bars represent the SD. *, P < 0.05; **, P < 0.01.

To study whether the transcription of *OTUB1* was regulated by ERRα, we constructed the *OTUB1* luciferase reporter by inserting the -1,000 to +1,000 region of the *OTUB1* gene upstream of the firefly luciferase coding sequence in the vector pGL3-Basic (Figure 2A). Luciferase activity assays showed that the *OTUB1* promoter was activated by ERRα overexpression in HCT116 and Caco-2 cells (Figure 2B and E). By contrast, knockdown of *ERRα* expression or inhibition of ERRα through XCT790 suppressed the promoter activity (Figure 2C, D, F and G).

**Figure 3:**
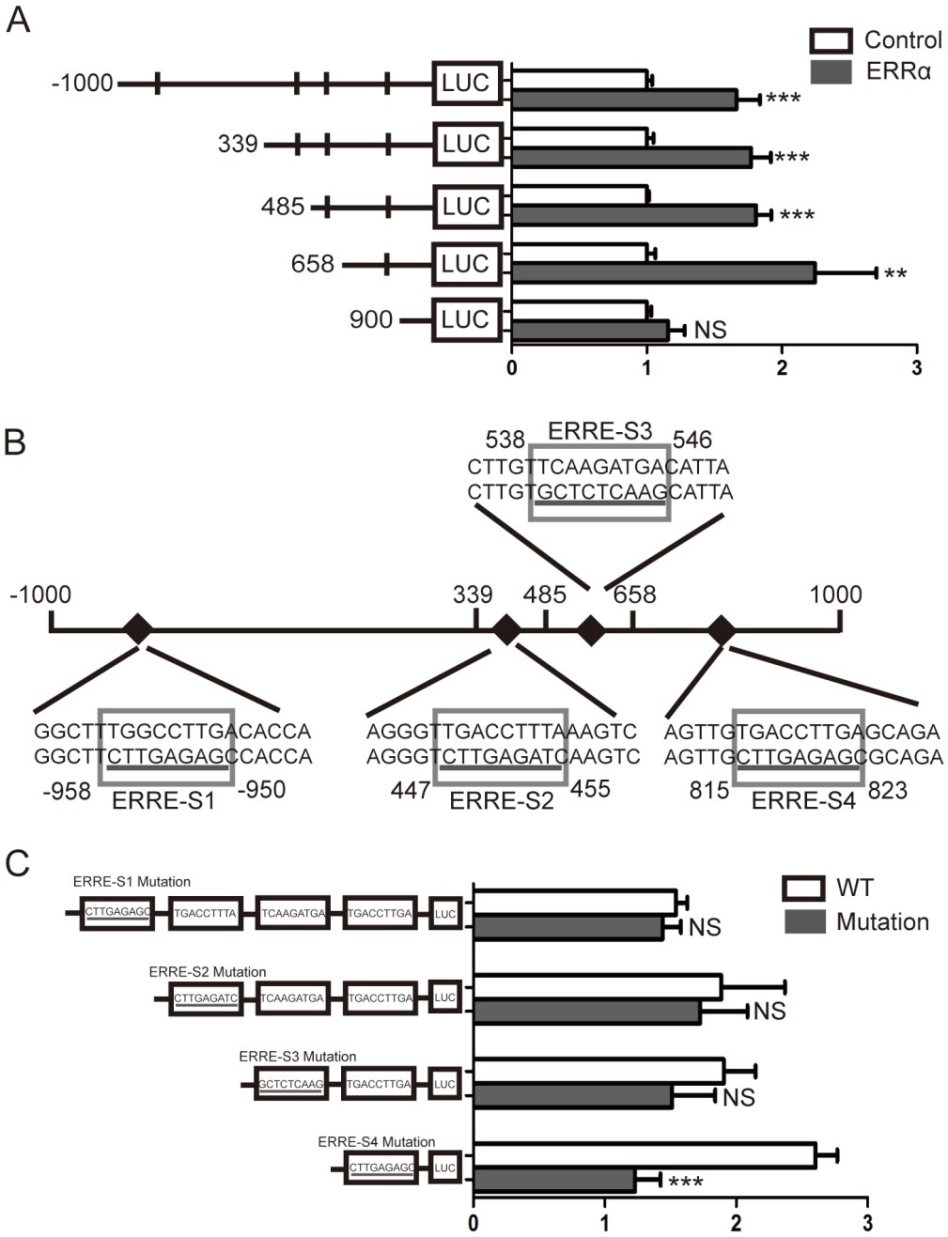
** Identification of putative ERRα binding sites on *OTUB1* promoter.** (A) An empty vector or an ERRα expression plasmid was co-transfected with constructs containing different lengths of *OTUB1* promoter (-1000/+1000, +339/+1000, +485/+1000, +658/+1000, +900/+1000) cloned in a luciferase reporter plasmid into HCT116 cells for 48 h. The cells were then lysed and assayed for luciferase activity. Data were normalized to Renilla activity. (B) Diagram of the *OTUB1* locus showing ERRE-S1~4 in the promoter and the mutation sequences. (C) An empty vector or ERRα expression plasmid was co-transfected with wild-type or mutated *OTUB1*-luciferase constructs into HCT116 cells for 48 h. The luciferase activity was then assayed. Data were normalized to Renilla activity. The data represent the mean of three independent experiments, and the error bars represent the SD. **, P < 0.01; ***, P < 0.001.

Four potential ERRα response elements (ERREs) were found within *OTUB1* promoter: ERRE-S1 (TGGCCTTGA), located at -958/-950 bp from the transcription initiation site; ERRE-S2 (TGACCTTTA) located at 447/455 bp; ERRE-S3 (TCAAGATGA) located at 538/546 bp; and ERRE-S4 (TGACCTTGA) located at 815/823 bp (Table S2). To determine which of these was bound by ERRα, a series of truncated *OTUB1* promoter constructs were generated and co-transfected with the ERRα expression vector or empty vector. We found that the activity of the constructs with the -1000/+1000, +339/+1000, +485/+1000, +658/+1000 *OTUB1* promoter sequences were similar, indicating that they all contained the ERRα binding site. However, the activity of the reporter with the +900/+1000 *OTUB1* promoter sequence was not increased by ERRα (Figure 3A). We also mutated the putative ERRα binding sites and, again, conducted luciferase reporter assays after their co-transfection with the ERRα expression vector or empty vector (Figure 3B). We found that the mutation of ERRE-S1, ERRE-S2 or ERRE-S3 did not affect the luciferase activity, while the mutation of ERRE-S4 caused a significant decrease in the luciferase activity, suggesting that ERRα binding site was indeed ERRE-S4.

**Figure 4:**
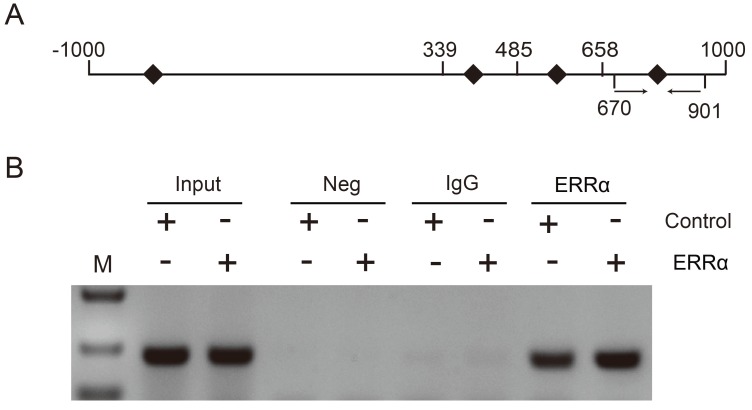
** ERRα binds to ERRE-S4 on *OTUB1* promoter.** (A) Schematic representation of the possible PCR products obtained after CHIP. (B) HCT116 cells were transfected with an empty vector or an ERRα expression plasmid for 48 h, and ChIP assays were performed using a human ERRα-specific antibody and the purified DNA was amplified by PCR. Input or negative control (NC) were not treated with antibody or were incubated with rabbit IgG, respectively.

To further demonstrate that ERRα binds to the *OTUB1* promoter through ERRE-S4, we conducted ChIP assays in HCT116 cells and used primers amplifying the +670/+901 region of *OTUB1* promoter, containing ERRE-S4 (Figure 4A). As shown in Figure 4B, ChIP assays showed a significant enrichment of the fragment that contained the ERRE-S4 site in the samples incubated with the anti-ERRα only. These results confirmed the interaction between ERRα and the ERRES4 site on *OTUB1* promoter.

### ERRα promotes CRC cell migration in an OTUB1-dependent manner

**Figure 5:**
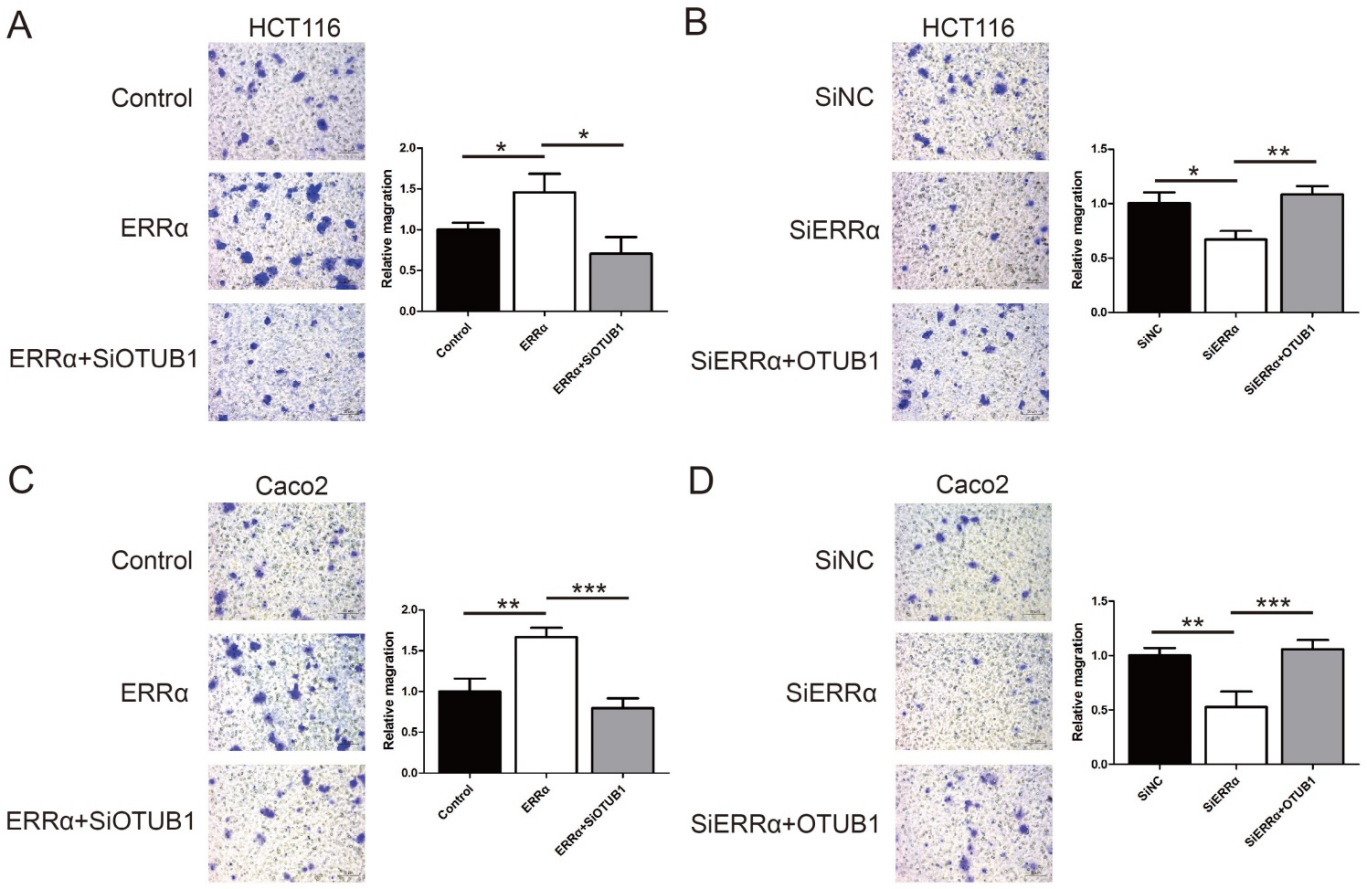
** ERRα promotes CRC cell migration via OTUB1**. HCT116 (A) or Caco2 (C) cells were transfected with ERRα expression plasmid (or empty vector) with or without OTUB1 siRNAs for 24 h. Then, 0.5 × 10^5^ HCT116 cells or 1.5 × 10^5^ Caco2 were seeded into a Boyden chamber for 24 to 36 h to assay cell migration. HCT116 (B) or Caco2 (D) cells were transfected with an ERRα siRNA (or siNC) with or without OTUB1 expression plasmid for 24 h. Then, 0.5 × 10^5^ HCT116 cells or 1.5 × 10^5^ Caco2 were seeded into a Boyden chamber for 24 to 36 h to assay cell migration. Representative images are shown. The relative magration of cells is indicated on the right. Bars represent the mean ± SD of three independent experiments. *, P < 0.05; **, P < 0.01; ***, P < 0.001.

To assess the function of ERRα and OTUB1 in CRC cell migration, transwell assays were performed. First, the effect of siRNAs of OTUB1 was tested by western blot (Figure S3). As show in Figure 5A and C, the migration ability of ERRα-overexpressing cells was enhanced, and the knockdown of* OTUB1* reversed this effect. In contrast, silencing of ERRα significantly suppressed the migration of HCT116 and Caco2 cells, and this effect was reversed by overexpression of OTUB1 (Figure 5B and D).

### ERRα promotes CRC cell migration through vimentin

**Figure 6:**
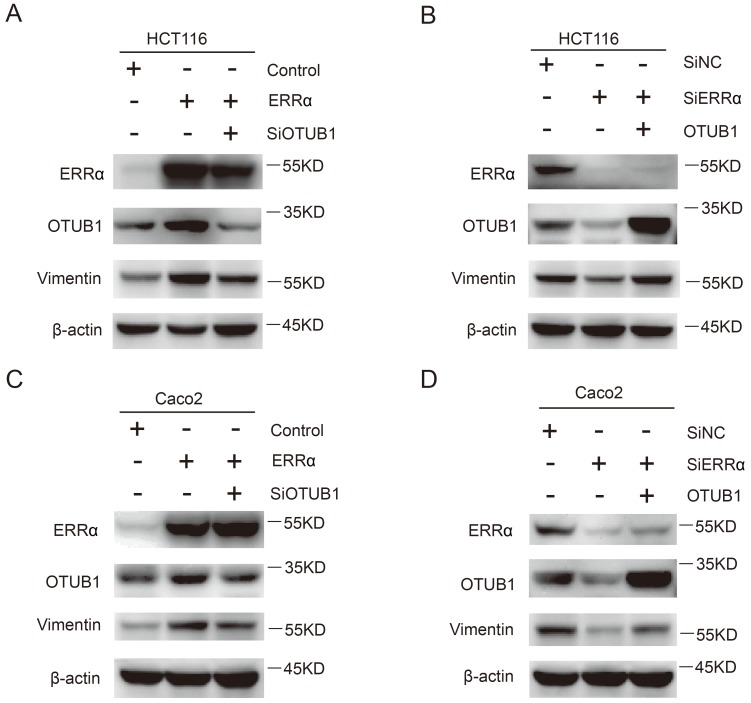
** ERRα promotes CRC cell migration through vimentin.** HCT116 (A) or Caco2 (C) cells were transfected with ERRα expression plasmid (or empty vector) with or without OTUB1 siRNAs for 48 h. The protein levels of ERRα, OTUB1 and vimentin were measured by western blot. HCT116 (B) or Caco2 (D) cells were transfected with an ERRα siRNA (or siNC) with or without OTUB1 expression plasmid for 48 h. The protein levels of ERRα, OTUB1, and vimentin were measured by western blot.

Finally, we investigated how the effect of ERRα on OTUB1 promotes CRC cells migration, which is essential for tumor metastasis. As show in Figure 6A and C, the expression of vimentin was increased in ERRα-overexpressing cells, this effect was counteracted by *OTUB1* knockdown. On the other hand, knockdown of *ERRα* expression significantly decreased vimentin levels and, again, this effect was reversed by OTUB1 overexpression (Figure 6B and D). However, we did not detect changes in the expression of E-cadherin, β-catenin, snail, and zo-1 (Figure S4). These results showed that ERRα regulates expression of OTUB1 and promotes colorectal cell migration through vimentin.

## Discussion

In this study, we demonstrated that ERRα regulates *OTUB1* expression by binding the *OTUB1* promoter and promotes migration of CRC cells through vimentin. Recent studies have reported the oncogenic role OTUB1 in many cancers, such as prostate, ovarian, breast and lung cancer, esophageal squamous cell carcinoma (ESCC), hepatocellular carcinoma (HCC), and CRC. OTUB1 mediates prostate cancer tumorigenesis and invasion through RhoA activation 24. Additionally, it promotes tumor progression and chemotherapy resistance by inhibiting FOXM1 degradation in ovarian cancer and breast cancer 25, 26. Moreover, in lung cancer, by inhibiting RAS ubiquitination, OTUB1 triggers cancer development 27. OTUB1 expression is also associated with a malignant phenotype in digestive tract cancer. In ESCC, OTUB1 predicts poor prognosis and facilitates metastasis stabilizing Snail and promoting EMT 28. OTUB1 is highly expressed in HCC, and promotes HCC cells proliferation and invasion 29. In gastric cancer, OTUB1 is an independent risk factor for disease-specific survival and enhances tumor invasiveness 30. In CRC, OTUB1 induces metastasis by regulating EMT 22. OTUB1 is overexpressed in prostate cancer, ovarian cancer, breast cancer, ESCC, HCC, gastric and CRC. However, the molecular mechanism underlying OTUB1 overexpression has not been clarified. Yuan and collogues have shown that the microRNA miR-542-3p targets OTUB1 and inhibits cell proliferation and invasion in CRC 31. In this study, we found that ERRα regulates OTUB1 expression in CRC by binding to *OTUB1* promoter.

ERRα has been shown to be important for cell metabolism, tumorigenesis, cancer proliferation and metastasis. ERRα is overexpressed in CRC tissues and high ERRα levels indicate poor prognosis 15, 16. By analyzing the TGCA database, we found that the expression of* OTUB1* is highly related to that of *ERRα*. ERRα is an orphan nuclear receptor which binds to TNAAGGTCA sequences on DNA. By binding to their promoters, ERRα can regulate many genes in colorectal cell lines, such as *Osteopontin* (*OPN*) in HT29 cells 32 and *SULT2A1* in Caco-2 cells 33. Here, we found four putative ERREs on *OTUB1* promoter. Overexpression or downregulation of ERRα in HCT116 and Caco2 cells increased or decreased promoter activity, and regulated OTUB1 expression. Deletion and mutation analyses of *OTUB1* promoter and CHIP assays revealed that ERRα regulates OTUB1 expression by binding to ERREE-S4 on *OTUB1*. Therefore, OTUB1 is a novel target of ERRα in CRC.

Previous studies have shown that ERRα promotes CRC cell proliferation by targeting a large set of key genes related to glycolysis, tricarboxylic acid cycle and lipid synthesis 34. Moreover, ERRα promotes the proliferation and migration of colorectal cells by upregulating IL-8 and thus inducing the phosphorylation of ERK1/2 and STAT3 35. In lung cancer, ERRα induces EMT by regulating Slug and promotes the migration and invasion of A549 cells 36. In triple-negative breast cancer, ERRα can directly bind the *fibronectin* promoter, not *Snail* or *Slug* promoters, and promote EMT 37. In this study, we demonstrated that ERRα targets *OTUB1* and promotes cells migration through vimentin. Vimentin is an intermediate filament protein that is expressed in mesenchymal cells and a marker for EMT. Vimentin overexpression in epithelial cells indicates enhanced migration ability. In CRC, high vimentin expression predicts poor disease-free survival and overall survival 38 and it is a biomarker for lymph node metastasis 39. In SW480 and SW620 cells models, OTUB1 can regulate EMT molecular markers such as E-cadherin and vimentin 22. However, in this model (HCT116 and Caco2 cells), ERRα upregulated only vimentin via OTUB1, but not other EMT markers.

## Conclusions

Our findings demonstrate that ERRα promotes the migration of CRC cells through OTUB1. By binding *OTUB1* promoter, ERRα regulates *OTUB1* expression and promotes EMT of colorectal cells. Therefore, targeting ERRα and OTUB1 might be a new strategy for CRC treatment.

## Supplementary Material

Supplementary figures and tables.

## Abbreviations

ERRα: estrogen-related receptor alpha
OTUB1: ovarian tumor domain-containing ubiquitin aldehyde binding protein 1
CRC: colorectal cancer
ESCC: esophageal squamous cell carcinoma
HCC: hepatocellular carcinoma
ERREs: ERRα response elements
CHIP: Chromatin Immunoprecipitation
