# Supplementary material for: ERRα Regulates OTUB1 Expression to Promote Colorectal Cancer Cell Migration
Source: J Cancer. 2019 Oct 6;10(23):5812–9. doi: 10.7150/jca.30720 (PMC6843886; doi:10.7150/jca.30720)
Supplement: Supplementary file 1 — Supplementary figures and tables. [file jcav10p5812s1.pdf]

Supplementary Figures

Supplementary Figure S1

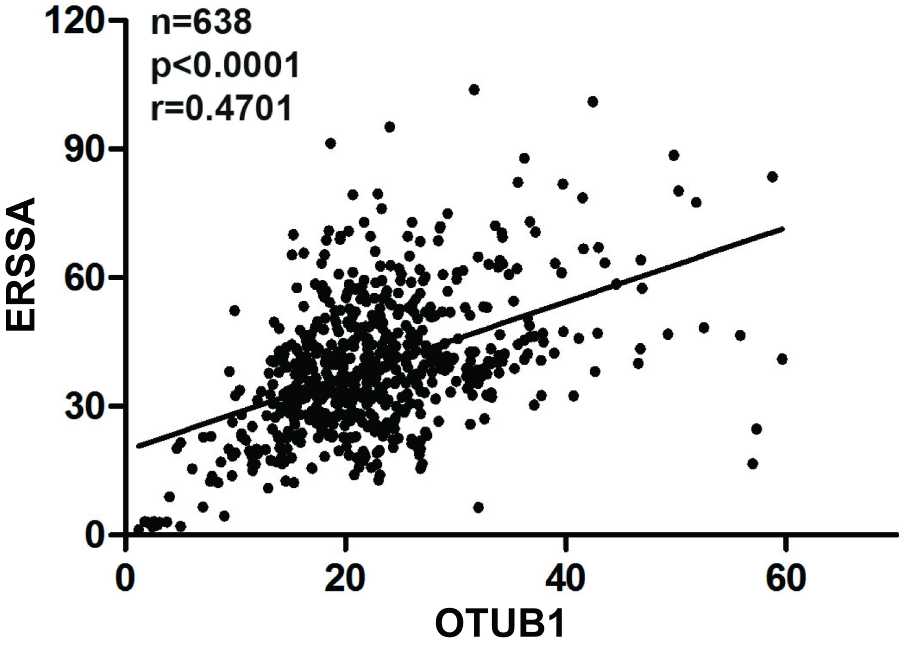

Figure S1. The expression of ERRα and OTUB1 correlate in CRC. Correlation analysis relative to the expression of ERRα and OTUB1 in 638 patients with CRC in the TCGA database.

Supplementary Figure S2

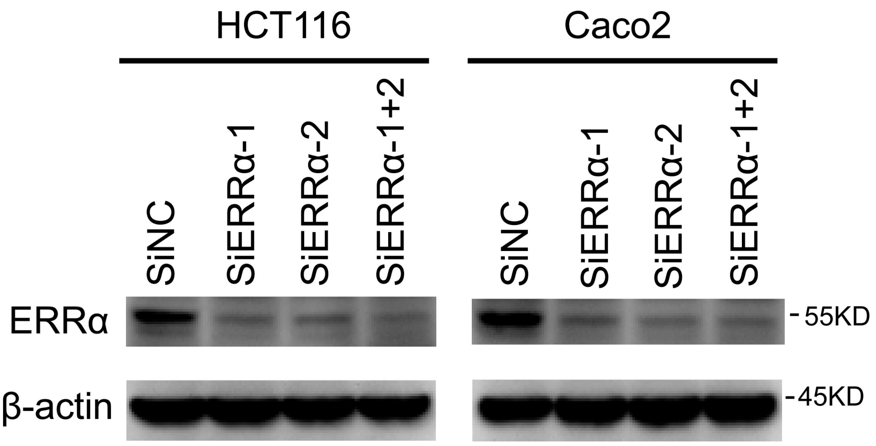

Figure S2. ERRα knock down by siRNA. Two siRNAs targeting ERRα were transfected alone or together into HCT116 or Caco2 cells for 48 h. ERRα levels were quantified by western blot.

Supplementary Figure S3

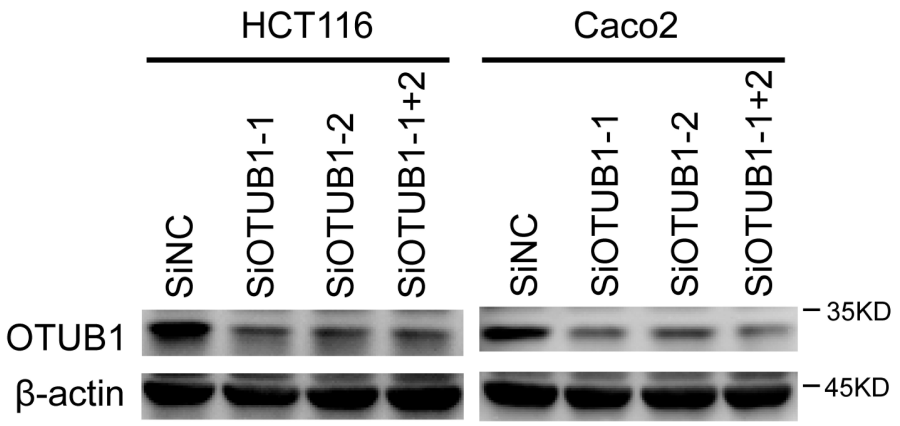

Figure S3. OTUB1 knock down by siRNA. Two siRNAs targeting OTUB1 were transfected alone or together into HCT116 or Caco2 cells for 48 h. OTUB1 were quantified by western blot.

Supplementary Figure S4

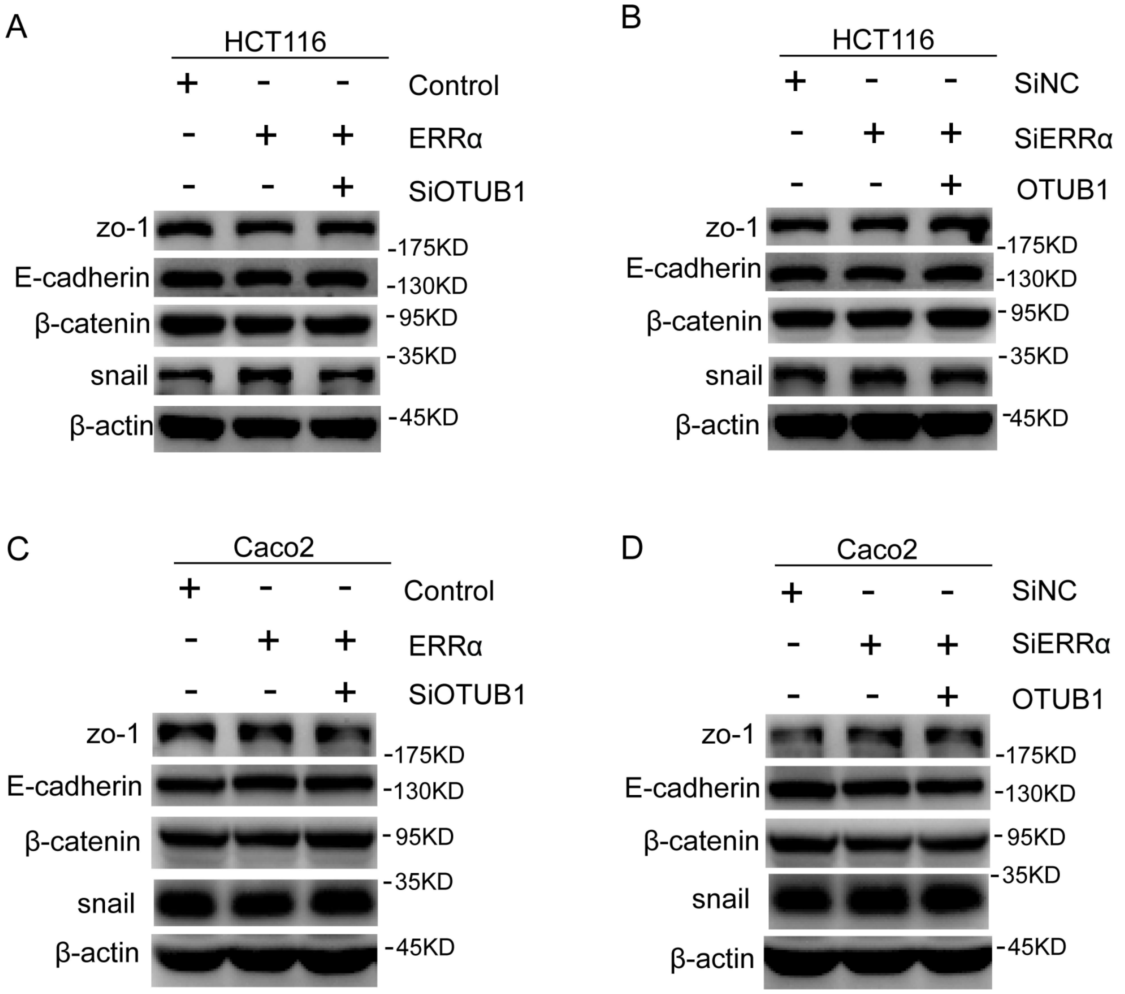

Figure S4. ZO-1, E-cadherin, β-catenin and snail were not regulated by ERRα. HCT116 (A) or Caco2 (C) cells were transfected with ERRα expression plasmid (or empty vector) with or without OTUB1 siRNAs for 48 h. HCT116 (B) or Caco2 (D) cells were transfected with an ERRα siRNA (or siNC) with or without OTUB1 expression plasmid for 48 h. The protein levels of ZO-1, E-cadherin, β-catenin, and snail were measured by

western blot.

Supplementary tables

Table S1. The primers were used for cloning OTUB1 promoter

promoter	primer	sequence
-1000/+1000	forward	5’-TATACGCGTAAACCAGAAGGACACAGA-3’
	reverse	5’-TCCAGATCTAGGCGTGGTCTCTTGCAG-3’
+339/+1000	forward	5’-TATACGCGTCCGCGTAGGTCATTGGGT-3’
	reverse	5’-TCCAGATCTAGGCGTGGTCTCTTGCAG-3’
+485/+1000	forward	5’-TATACGCGTACCGAACACTTGAGGGAA-3’
	reverse	5’-TCCAGATCTAGGCGTGGTCTCTTGCAG-3’
+658/+1000	forward	5’-TATACGCGTCGTACTGGTATGCCTCTA-3’
	reverse	5’-TCCAGATCTAGGCGTGGTCTCTTGCAG-3’
+900/+1000	forward	5’-TATACGCGTGGATGAAGGCGGTGATGGA-3’
	reverse	5’-TCCAGATCTAGGCGTGGTCTCTTGCAG-3’
ERRE-S1 mutation	Forward-1	5’-TATACGCGTAAACCAGAAGGACACAGA-3’
	Reverse-1	5’-ATCCCTTGGTGGCTCTCAAGAAGCCTCATGACCTAACT-3’
	Forward-2	5’-GGCTTCTTGAGAGCCACCAAGGGATGCTGTCT-3’
	Reverse-2	5’-TCCAGATCTAGGCGTGGTCTCTTGCAG-3’
ERRE-S2 mutation	Forward-1	5’-TATACGCGTCCGCGTAGGTCATTGGGT-3’
	Reverse-1	5’-GACTTGATCTCAAGACCCTTTTGGGGCTCGCC-3’
	Forward-2	5’-AGGGTCTTGAGATCAAGTCATTCAGCCCACGC-3’
	Reverse-2	5’-TCCAGATCTAGGCGTGGTCTCTTGCAG-3’
ERRE-S3 mutation	Forward-1	5’-TATACGCGTACCGAACACTTGAGGGAA-3’
	Reverse-1	5’-TAATCTTGAGAGCCAAGTCCCTTCCGGGGC-3’
	Forward-2	5’-CTTGTGCTCTCAAGCATTAAGAGAGAGCCAGG-3’
	Reverse-2	5’-TCCAGATCTAGGCGTGGTCTCTTGCAG-3’
ERRE-S4 mutation	Forward-1	5’-TATACGCGTCGTACTGGTATGCCTCTA-3’
	Reverse-1	5’-TCTGCGCTCTCAAGCCAACTAGCAAAAGACCTGC-3’
	Forward-2	5’-AGTTGCTTGAGAGCGCAGATTGCTTCACCTGTC-3’
	Reverse-2	5’-TCCAGATCTAGGCGTGGTCTCTTGCAG-3’

Table S2. The potential ERRα response elements of OTUB1 promoter −1000 to +1000

Model name	Score	Relative score	Start	End	Strand	predicted site sequence
ESRRA	14.251	0.924712	815	823	-1	TCAAGGTCA
ESRRA	11.482	0.890292	447	455	-1	TAAAGGTCA
ESRRA	8.989	0.859303	-958	-950	-1	TCAAGGCCA
ESRRA	4.546	0.804075	538	546	1	TCAAGATGA
